# ARF6 and Rab11 as intrinsic regulators of axon regeneration

**DOI:** 10.1080/21541248.2018.1457914

**Published:** 2018-05-17

**Authors:** Bart Nieuwenhuis, Richard Eva

**Affiliations:** a John van Geest Centre for Brain Repair, Department of Clinical Neurosciences, University of Cambridge, Forvie Site, Robinson Way, Cambridge, UK; b Laboratory for Regeneration of Sensorimotor Systems, Netherlands Institute for Neuroscience, Royal Netherlands Academy of Arts and Sciences (KNAW), Amsterdam, The Netherlands

**Keywords:** ARF6, axon transport, axon regeneration, integrins, polarised transport, Rab11, spinal cord injury

## Abstract

Adult central nervous system (CNS) axons do not regenerate after injury because of extrinsic inhibitory factors, and a low intrinsic capacity for axon growth. Developing CNS neurons have a better regenerative ability, but lose this with maturity. This mini-review summarises recent findings which suggest one reason for regenerative failure is the selective distribution of growth machinery away from axons as CNS neurons mature. These studies demonstrate roles for the small GTPases ARF6 and Rab11 as intrinsic regulators of polarised transport and axon regeneration. ARF6 activation prevents the axonal transport of integrins in Rab11 endosomes in mature CNS axons. Decreasing ARF6 activation permits axonal transport, and increases regenerative ability. The findings suggest new targets for promoting axon regeneration after CNS injury.

## Selective polarised transport and axon regeneration

**Figure 1.**
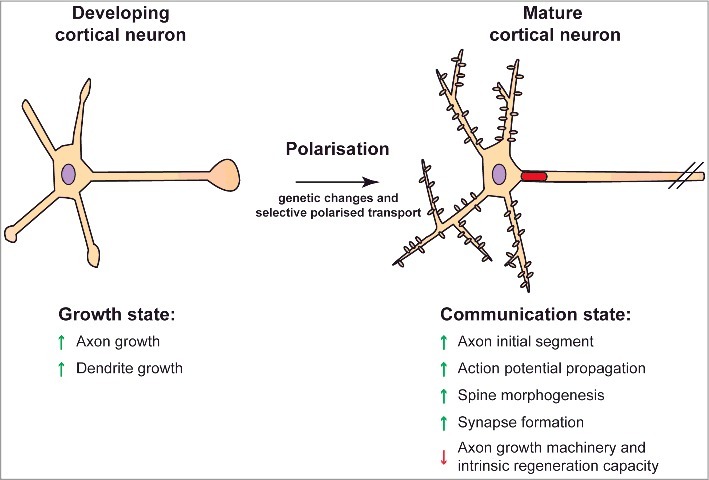
Polarisation of cultured cortical neurons. Neurons undergo various developmental stages during polarisation. Genetic changes and selective transport of proteins contribute to neuronal polarisation. For simplification purposes, two developmental stages are categorized here. Developing cortical neurons exist in a growth state that promotes first axonal and then dendritic outgrowth. As neurons mature, there is a decline in their growth capacity as they become geared for neurotransmission. The neurons form an axon initial segment that propagates the action potential and also contributes to polarised membrane protein transport. Mature cortical neurons have a poor intrinsic capacity for axon regeneration.

Axons in the adult central nervous system (CNS) are particularly susceptible to injury and insult. This is exacerbated by the fact that after they have been damaged, adult CNS neurons do not regenerate. Injuries to the brain and spinal cord can therefore have serious life changing consequences. Neurons are highly polarised cells with multiple dendrites and a single axon. In adulthood, the axon propagates the action potential and releases neurotransmitters, whilst dendrites receive and process information from the numerous axonal terminals that synapse on them. For these diverse functions, axons and dendrites require different intracellular machinery. This demands precise delivery and/or retention of the correct molecules to their required locations. Membrane protein targeting is particularly precise, being regulated by numerous signalling, trafficking and transport pathways [1]. This ability of neurons to selectively transport the correct molecules to either axons or dendrites is termed selective polarised transport. Crucially, this process changes with development of the neuron. In developing neurons, the machinery required for axon growth is abundantly transported towards the tip of the growing axon – the growth cone. This includes numerous cell-surface membrane proteins such as growth factor and guidance receptors, as well as the cytosolic machinery required for the growth process, including cytoskeletal machinery and accompanying regulatory molecules, and the traffic and transport molecules required for membrane addition and reorganisation [2]. As neurons mature, the role of the axon changes towards electrical excitability and neurotransmission. Axon growth machinery is no longer required, and presynaptic membrane trafficking processes become specialised for synaptic vesicle recycling and release (exocytosis) (Figure 1).

It is now becoming apparent that one of the consequences of this developmental alteration in selective polarised transport is that adult CNS axons do not possess sufficient growth-enabling machinery for regeneration after an injury [3,4]. This may be one of the reasons that CNS axons are particularly vulnerable to insult and damage, and why injury to axons in the adult brain or spinal cord has such devastating consequences. Two recently published papers describe roles for Rab11 and ARF6 in the regulation of selective polarised transport and axon regeneration in mature CNS neurons [5,6]. ARF6 and Rab11 are small GTPases that regulate the traffic and transport of recycling endosomes. The two papers demonstrate that as CNS axons mature, ARF6 functions to remove axon growth machinery (integrins in Rab11 endosomes) from axons, constraining it to the somato-dendritic domain. This mini-review summarises the findings of the two papers, providing some background and discussing the implications for future research into novel treatments for promoting axon regeneration after spinal cord injury.

## Factors preventing axon regeneration after spinal cord injury

There are a number of events that prevent repair after spinal cord injury, but essentially, axon regeneration fails for two key reasons: extrinsic inhibitory molecules prevent attempts at regrowth, and adult CNS axons have a poor intrinsic capacity for regeneration [7,8]. Axons descending from the brain through the corticospinal tract (CST) are responsible for voluntary motor control, so when these are injured, paralysis occurs. Research is therefore aimed at enabling CST axons to regenerate past the injury site to make new connections leading to restoration of motor function. An optimal repair strategy will enable CST axons to regenerate over long distances, and allow them to re-establish their correct connections. Injury to the spinal cord leads to the formation of the glial scar. During this process, a number of inhibitory molecules are laid down around the site of injury. These have been well characterised, and include myelin associated molecules such as NogoA, MAG, and OMgp as well as astrocyte derived molecules such as the chondroitin sulphate proteoglycans (CSPGs). Targeting some of these inhibitory molecules has generated successful interventions that can stimulate axonal sprouting and plasticity leading to partial recovery of function, and these discoveries are now progressing towards clinical treatments [9, 10]. However, overcoming extrinsic inhibition has not led to long-range axon regrowth. More recent studies have therefore been aimed at increasing the axon's intrinsic regenerative ability, and these have identified signalling pathways and transcription factors that can be targeted to stimulate meaningful regeneration [8,11–13], but these approaches have not enabled long-range regeneration of CST axons past the site of injury. There are therefore continued efforts into identifying means of enabling robust axon regrowth of CST axons over long distances through the adult spinal cord.

## Integrins can enable long-range regeneration in the spinal cord

Long-range regeneration is possible in the spinal cord, as has been demonstrated for sensory neurons regenerating their axons towards the brain. Sensory axons normally extend from the peripheral nervous system (PNS) into the spinal cord to relay sensory information. Some sensory axons form synapses and terminate in the spinal cord, whilst others continue towards the brain to form connections in the medulla [14]. After an injury, sensory neurons can regenerate their axons but their growth is usually halted by inhibitory molecules in the spinal cord, including the extra-cellular matrix (ECM) molecule, tenascin-C. Long-range regeneration of sensory axons was enabled by virally transducing them with an integrin that recognises tenascin-C, alpha9beta1 [15]. Integrins are a complex family of hetero-dimeric cell surface receptors which recognise molecules within the ECM governed by their alpha-beta combinations [16]. Integrins mediate guided CNS axon growth during development and in the PNS they can drive axon regeneration after injury [17,18]. Viral expression of alpha9 integrin together with its activator kindlin-1 endows sensory axons with the ability to ignore inactivation by inhibitory molecules, leading to robust regeneration from the level of the forelimb to the medulla. This method enables re-establishment of connections within the spinal cord, leading to functional sensory recovery [15]. The approach works because PNS axons efficiently transport integrins, allowing them to guide axon regeneration from the axon surface. The strategy could be used to enable regeneration of descending motor axons in the CST, but crucially, integrins are restricted from these axons. Endogenous integrins are not transported into adult CNS axons, being found in dendrites and the cell body [19,20], and viral delivery of alpha9 integrin into adult CNS neurons similarly allows transport of integrins into dendrites but not into axons [3]. Importantly, integrins are transported into immature, developing axons in the CNS, and these axons have a greater regenerative capacity.

It is therefore likely that the restriction of integrins from adult CNS axons contributes to their inability to regenerate. The papers described in this review were aimed at understanding the mechanisms controlling axonal integrin traffic and transport, with a view to identifying means of directing integrins into mature CNS axons. The authors reasoned that this might raise their regenerative ability. Additionally, it might also mean that the integrin method which enables robust sensory regeneration through the spinal cord could be applied to the CST axons that control motor function. It should also clarify whether the CNS blockade of integrin axon transport contributes to regenerative failure.

## Rab11 and ARF6

**Figure 2.**
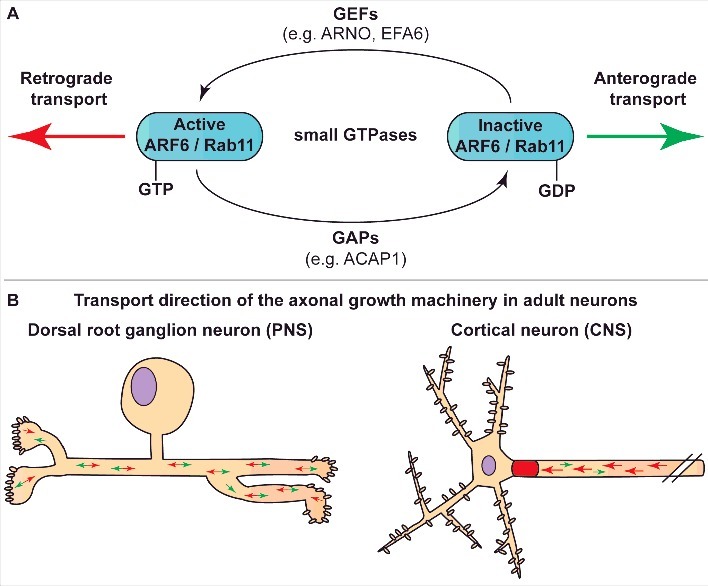
The activation state of small GTPases ARF6 and Rab11 regulate the transport direction of the axonal growth machinery. A. Molecular mechanisms of GTPases activation and inactivation. Active, GTP-bound ARF6 / Rab11 stimulate retrograde transport *via* dyneins, whilst GDP bound ARF6 / Rab11 favours anterograde transport *via* kinesin motors. Guanine nucleotide exchange factors (GEFs), such as ARNO and EFA6, activate GTPases by exchanging GDP for GTP and thereby promote retrograde transport. GTPase-activating proteins (GAPs), including ACAP1, stimulate GTP to GDP hydrolysis and promote anterograde transport of kinesins. B. Summary of the transport direction of the axonal growth machinery in adult dorsal root ganglion (DRG) and cortical neurons. Vesicles containing integrins move in bi-directional direction in DRG, while cortical neurons exhibit predominant retrograde transport of axonal growth machinery away from the axon. Green arrow indicates anterograde transport, red arrow indicates retrograde transport. PNS, peripheral nervous system; CNS, central nervous system.

Previous studies on integrin traffic in PNS axons found that integrins are transported in recycling endosomes controlled by the small GTPases, Rab11 and ARF6. Rab11 targets integrins to the axonal growth cone surface, and functions at the growth cone to regulate integrin recycling [21], whilst ARF6 controls the direction of axonal integrin transport [22]. Active ARF6 stimulates retrograde transport, whereas inactive ARF6 allows anterograde transport. In PNS axons, integrins are efficiently transported and move bi-directionally, but in mature CNS axons they are removed by predominant retrograde transport controlled by mechanisms involving ARF6 activation and the axon initial segment [20] (Figure 2).

Rab11 and ARF6 have overlapping and distinct roles in the regulation of membrane protein trafficking through recycling endosomes. Rab11 was originally identified as a regulator of traffic from recycling endosomes towards the cell surface via the peri-nuclear recycling centre [23]. Subsequently it was found that Rab11 is also involved in more rapid forms of localised recycling such as the high capacity turn-over of membrane proteins that is observed in migrating cells [24], as well as being involved in a wider range of trafficking pathways, including exocytosis [25]. It's role in axon growth is now well accepted, being responsible for the transport and recycling of integrins and TrkA in PNS axons and growth cones [21,26] as well as having a developmental role in the CNS, where it is involved in the regulated insertion of guidance receptors when developing axons cross the ventral midline of the spinal cord [27]. Importantly, Rab11 appears to be essential for growth cone function because its targeted removal (by optogenetics) leads to growth cone collapse [28]. It is also known to regulate the trafficking of a number of molecules which can stimulate axon growth in addition to integrins and Trk receptors, including the EGF, IGF and FGF receptors [29-31] and the pro-regenerative reggie/flotillin molecules [32] (Table 1). In addition to these molecules, Rab11 also regulates the process of neurite outgrowth by supply the materials required for neurite extension through interactions with other Rab proteins such as Rabs 8 and Rab10 [33].

**Table 1.** Rab11 as an intrinsic regulator of axon growth and regeneration. This table consists of two sections demonstrating that Rab11 is important for axon growth and regeneration. The first section (grey, left columns) highlights the regeneration-associated proteins localised in Rab11-positive endosomes. The second section (white, right columns) summarises the main findings regarding axon growth and regeneration of the mentioned proteins. Studies that investigated neurite outgrowth in neuronal cell lines were excluded from this table.

Regeneration-associated proteins in Rab11-positive-endosomes	References	Main findings regarding axon growth and regeneration	References
Rab11 (GTPase)	Not applicable	Expression enhanced the axon regeneration capacity of cortical neurons after *in vitro* laser axotomy	Koseki *et al.*, (2017)
Integrins	Caswell *et al.*, (2008);	Expression of α9 integrin promoted dorsal root ganglia regeneration in rat	Andrews *et al.,* (2009); Cheah *et al.,* (2017)
	Eva *et al.*, (2010)	Genetic deletion of α7 integrin impaired facial nerve regeneration in mice	Werner *et al.,* (2000)
		Genetic deletion of α7 integrin impaired sciatic nerve regeneration in mice	Gardiner *et al.,* (2005)
Insulin-like growth factor receptors (IGFRs)	Romanelli *et al.*, (2007)	Function-blocking antibody of IGF-1R impaired axon outgrowth of corticospinal motor neuron in postnatal mice	Ozdiner *et al.,* (2006)
		*In utero* shRNA interference of IGF-1R impaired neuronal migration and axon formation in embryonic/postnatal mice	Guil *et al.,* (2017)
Reggies/flotillins	Solis et al., (2013); Huelsbusch et al., (2015);	Expression promoted retinal ganglion cells regeneration in rat	Koch *et al.,* (2013)
	Bodrikov et al., (2017)	Morpholino interferences impaired retinal ganglion cells regeneration in zebrafish	Munderloh *et al.,* (2009)
Tropomyosin receptor kinase receptors (Trks)	Ascaño *et al.*, (2009);	Expression of TrkB promoted corticospinal motor neuron regeneration after subcortical axotomy in rat	Hollis *et al.*, (2009)
	Lazo *et al.*, (2013)		

ARF6 also regulates endocytic traffic through the recycling pathway, and is additionally involved in the regulation of the actin cytoskeleton and phosphoinositide signalling. As it has very low intrinsic GTPase activity, ARF6 is strongly reliant on its GAPs and GEFs for regulation of nucleotide cycling [34]. ARF6 and its regulatory molecules have been closely linked to the control of integrin traffic and function, regulating integrin traffic in migrating and invasive cells, as well as controlling the function of integrin adhesion complexes [35]. Our own early studies found a role for ARF6 in the regulation of integrin traffic in PNS axons, and other work has demonstrated a role for ARF6 in the regulation of developmental axon growth in CNS neurons, partly as a result of signalling through phosphoinositides [36] (Table 2). ARF6 also plays a clearly defined role in the regulation of neurite outgrowth in PC12 cells [37], as well as regulating the migration of neurons in the cerebral cortex during development functioning via the ARF6 and Rab11 effector (FIP3)/Arfophilin-1 [38].

**Table 2.** ARF6 as an intrinsic regulator of axon growth and regeneration. This table highlights the effects of ARF6 and its associated GAP and GEFs on axon growth and regeneration *in vitro*. Stimulation of anterograde transport of the axonal growth machinery promotes growth, while retrograde transport hinders growth.

ARF6 GEF/GAP	Main findings regarding axon growth and regeneration	References
ARF6 (GTPase)	Expression of wild type ARF6 inhibited axon growth by 30% in developing cortical neurons *in vitro*	Suzuki *et al.,* (2010)
	Expression of wild type ARF6 or constitutively active ARF6 did not affect axon growth in developing hippocampus neurons *in vitro*	Hernandez-Deviez *et al.,* (2004)
	Expression of dominant negative ARF6 increased axon growth by 100% in developing hippocampus neurons *in vitro*	Hernandez-Deviez *et al.,* (2004)
	Expression of dominant negative ARF6 increased axon growth by 67% in developing cortical neurons *in vitro*	Suzuki *et al.,* (2010)
ACAP1 (GAP)	Expression promoted axon growth by 25% in adult DRG neurons *in vitro*	Eva *et al.,* (2012)
ARNO (GEF)	Expression of wild type ARNO inhibited axon growth by 50% in adult DRG neurons *in vitro*	Eva *et al.,* (2012)
	Expression of wild type ARNO did not affect axon growth in developing hippocampus neurons *in vitro*	Hernandez-Deviez *et al.,* (2004)
	Expression of catalytically inactive ARNO increased axon growth by 500% in developing hippocampus neurons *in vitro*	Hernandez-Deviez *et al.,* (2004)
	Expression of catalytically inactive ARNO promoted axon growth by 30% in cortical neurons *in vitro*	Franssen *et al.,* (2015)
EFA6 (GEF)	Expression inhibited axon growth by 50% in adult DRG neurons *in vitro*	Eva *et al.,* (2012)
	Expression inhibited the axon regeneration capacity of adult DRG neurons by 70% after *in vitro* laser axotomy	Eva *et al.,* (2017)
	shRNA interference increased the axon regeneration capacity of cortical neurons by 110% after *in vitro* laser axotomy	Eva *et al.,* (2017)

ARF6 also functions (in its active, GTP-bound state) in adult CNS neurons to limit axon growth by its ability to remove integrins from axons via retrograde transport, because overcoming this process increases developmental axon length [20]. It is likely that the control of directional transport within axons occurs through an interaction of ARF6 with the JIP3/4 scaffold molecules, although this has not been demonstrated in neurons. The ARF6 JIP3/4 interaction has been shown to control the direction of endosomal transport during cytokinesis, with ARF6-GTP increasing the affinity of JIP3/4 for the dynein/dynactin complex, leading to retrograde transport, whilst ARF6-GDP favours interactions with kinesin allowing anterograde transport along microtubules. This occurs in a complex involving both ARF6 and Rab11 [39]. In summary, Rab11 and ARF6 are recycling endosome markers that regulate integrin traffic and function and as well as developmental axon growth (Figure 2, and Tables 1 and 2).

## Rab11 and axon regeneration

To investigate whether Rab11 and ARF6 are involved in the regulation of axon regeneration in mature CNS axons, we established an *in vitro* model for studying axon regeneration [6]. The *in vitro* laser axotomy model provides a platform for the identification and validation of potential axon regeneration targets that could be taken forward for investigation in animal models of spinal cord injury. Rat cortical neurons were cultured to maturity (up to 24 days in vitro (DIV)) with an astrocyte feeder layer. This type of culture has been used before to investigate changes that occur during neuronal polarisation [40], but not in the context of axon regeneration. The cell cultures were validated by measuring electrical activity and gene changes at 4, 8, 16 and 24 DIV. The neurons underwent a developmental change in gene expression, showing an increase in genes involved in synapse formation, and exhibited increased electrical activity as the neurons matured. Importantly, ingenuity pathway analysis also revealed there was a decline in development-associated genes over time (see also Figure 1). To measure the intrinsic axon regeneration capacity, we used *in vitro* laser axotomy, which allows precise severing of individual axons. This confirmed that regenerative capacity declined in line with maturity [6]. We then used this *in vitro* model of regenerative decline to investigate a role for Rab11 in CNS axon regeneration. Our hypothesis was that the developmental decline in regenerative ability might be due to changes in selective polarised transport which restrict post-synaptic molecules to the somato-dendritic domain, limiting the molecules entering the axon. A number of studies have demonstrated that Rab11 is an important dendritic, post-synaptic molecule, being responsible for AMPA receptor recycling into dendritic spines [32], as well as trafficking the BDNF receptor, TrkB [41]. Rab11-positive-endosomes carry many cell-surface receptors that are important for axonal regeneration (Table 1), but has a somato-dendritic distribution in adult brain *in vivo* [42]. This was found to be the same in mature neurons *in vitro*. Importantly, Rab11 is present in equal amounts in axons and dendrites at an early stage (4 DIV), but by 16 DIV it is principally found in the cell body and dendrites, with low levels present in axons. To determine if this deficit of axonal Rab11 contributes to regenerative decline, we overexpressed Rab11 and measured its effects on axon regeneration. Overexpressed Rab11 is mis-trafficked into axons, indicating that the mechanism involved in restricting Rab11 to dendrites and the cell body is to a certain extent “leaky” after gene overexpression. Overexpression of Rab11 increased the regeneration potential and length of cortical neurons after laser axotomy. Importantly, overexpressing Rab11 also led to an increase in the amount of integrins present in axons, suggesting that increasing axonal Rab11 enhances regenerative capacity partly by providing the axon with regenerative machinery (integrins). This study demonstrated that cortical neurons can be used to investigate the intrinsic decline in axon regeneration ability that occurs with maturation in the CNS, and that the decline is partly due to the axonal exclusion of growth-promoting machinery in Rab11 endosomes.

## ARF6 and axon regeneration

ARF6 and Rab11 have overlapping and distinct roles, sometimes functioning as a complex [39], whist still being capable of regulating different subcellular events. This is the case in CNS neurons, because whilst ARF6 and Rab11 cooperate in the regulation of axonal integrin traffic, they also localise differently. Rab11 adopts a mainly somato-dendritic localisation, and functions principally in the post-synapse, whilst ARF6 appears to be uniformly distributed between axons and dendrites [20], having roles in both the pre- and post-synaptic compartments [43,44]. Our earlier studies found that ARF6 activation can control the direction of axonal integrin transport, and that two seemingly distinct mechanisms involving ARF6 activation and the axon initial segment (AIS) are responsible for the retrograde removal of integrins from mature CNS axons [20]. We reasoned that the two mechanisms might be linked, and the presence of an ARF6 GEF in the AIS would unite them. Looking again in cultured cortical neurons, we found that the ARF6 GEF EFA6 localises to the AIS as neurons mature, being undetectable at early time points but strongly enriched by 14 DIV onwards [5]. Our hypothesis was that EFA6 might be responsible for the retrograde removal of integrins from axons through axonal ARF6 activation. If this was the case, targeting EFA6 might facilitate integrin and/or Rab11 transport into axons (through the known interaction of Rab11 with ARF6), and this could subsequently enhance the axon's regenerative ability. To investigate whether EFA6 was activating ARF6 in the AIS we measured ARF activation using a GST tagged probe that binds only to active ARF (the ARF-binding domain of GGA3 fused to a GST tag). ARF protein activation was not restricted to the AIS, but was strongly present throughout the axon. Importantly, this was not evident earlier in development, when integrins and Rab11 are transported into axons. Silencing EFA6 sharply reduced axonal ARF activation, indicating that EFA6 activates ARF6 in mature CNS axons. The decreased ARF activation mediated by silencing EFA6 led to a decrease in retrograde transport and a substantial increase in anterograde axonal integrin transport. Crucially, silencing EFA6 also led to an increase in Rab11 endosomes in axons, most likely because Rab11 and ARF6 cooperate to control the direction of endosomal transport through interaction with scaffolds and motor proteins (although this remains to be tested in neurons). EFA6 therefore functions to remove axon growth machinery (integrins in Rab11 endosomes) from CNS axons as they mature.

To address whether this contributes to the maturational decline in regenerative capacity, we used the laser axotomy model to injure axons of neurons in which EFA6 had been silenced. Knockdown of EFA6 led to a substantial increase in regeneration after laser axotomy, suggesting that the GEF EFA6 contributes to the axon's poor regenerative ability. The findings suggest that ARF6 is an intrinsic regulator of regeneration, governed by its activation state. To investigate this idea further, experiments were performed to test whether increasing ARF6 activation would prevent PNS neurons from regenerating their axons after a laser injury. Adult dorsal root ganglia (DRG) neurons are sensory, PNS neurons with a superior regenerative ability compared to adult CNS neurons. As DRGs have a relatively good regeneration capacity, the hypothesis was that they either expressed less EFA6 than CNS neurons or that EFA6 is differently localised. Surprisingly, EFA6 was found at high levels in adult DRG neurons, although it was not enriched in the axon. Furthermore, PNS ARF6 activation was found to be counterbalanced by an ARF6 GAP that is not present in CNS neurons, ACAP1. Overexpressing EFA6 in DRG neurons resulted in expression throughout the axon, which led to a strong reduction in regeneration after laser injury. Expressing the ARF6 activation-incompetent EFA6 (EFA6 E242K) caused a much smaller reduction in regeneration. This further suggests that EFA6 opposes regeneration, functioning mostly through activation of ARF6. Taking the results from the experiments in CNS and PNS neurons together, we conclude that ARF6 activation state is an intrinsic determinant of regenerative capacity.

## Discussion – implications and future research

Overall, the two papers described suggest that ARF6 and Rab11 are both intrinsic regulators of regenerative capacity, and that a supply of growth promoting machinery in recycling endosomes is an important pre-requisite for re-establishing a growth cone which can enable vigorous axon growth. The cellular mechanisms required for growth cone development and axon growth have been well characterised, but it is not understood why these cannot be recapitulated after an injury to enable axon regeneration [45]. Investigations into the intrinsic mechanisms regulating axon regeneration have so far focused on upstream signalling molecules and transcription factors that can be targeted to facilitate regeneration. These have identified signalling through the PI3 kinase / PTEN pathway [46] and cytokine signalling pathways [47], and transcription factors such as sox11 [48] and KLF7 [13]. These pathways enhance regeneration through regulation of either transcription or translation, but importantly, it is not known how this results in axon growth. In other words, the downstream cellular mechanisms involved in mediating the actions of these regenerative interventions have not been characterised in detail. Our studies indicate that an efficient supply of axonal growth machinery is a critical factor determining an axon's regenerative ability. It will be important to discover whether axon transport is a mechanism that is involved downstream of any of the known regeneration interventions described above.

One pathway that is likely to function through the mobilisation of growth machinery is the PI3K / PTEN pathway. The tumour suppressor PTEN was identified as in intrinsic inhibitor of CNS regeneration almost 10 years ago [46], and subsequent studies have demonstrated that it is part of an important pathway controlling the regenerative capacity of both spinal cord and optic nerve axons. PTEN and PI3K transduce the actions of growth factor receptors and integrins. PI3K and PTEN therefore regulate two key signalling molecules, PIP_2_ and PIP_3_. PI3K generates the phosphatidylinositol PIP_3_ from PIP_2_, whilst PTEN converts PIP_3_ back to PIP_2_. Importantly, the complete mechanisms through which PTEN deletion promotes regeneration have yet to be fully characterised. One possibility is that PTEN may function partly by signalling via ARF6. The majority of ARF6 GEFs and GAPs are regulated either directly by PIP_2_ or PIP_3_, or by phosphorylation by kinases acting downstream of these phosphoinositides [49–51]. EFA6 is a good example of an ARF6 GEF that acts downstream of PI3K/PTEN, because it is strongly activated by PIP_2_ [52]. It remains to be seen whether elevating PI3K activity and therefore levels of PIP_3_ can overcome the activity of EFA6 (by converting PIP_2_ to PIP_3_) to promote axonal transport, but there is evidence that axonal signalling through PI3K can lead to increased anterograde transport of growth factor receptors in a feed forward fashion [53].

Further work is needed to ascertain whether EFA6 is a valid target for enhancing regeneration after a CNS axonal injury. To begin with it will be necessary to determine whether knock-down of EFA6 can enable integrin or Rab11 axonal transport into CNS axon *in vivo*. If this is the case, then it may be possible to apply the integrin method, (which has been used to promote sensory regeneration in the spinal cord [15]), in conjunction with EFA6 silencing, to achieve the goal of long-range regeneration of CST axons after a spinal cord injury. Alternatively, it may be necessary to intervene in additional ways, such as the overexpression of ARF6 GAPs, in order to overcome the predominant retrograde axonal transport which prevents an axonal integrin presence. Careful *in vivo* experiments are necessary to identify the optimal intervention for enabling integrin and Rab11 transport into mature CNS axons in animal models of CNS axonal injury.
